# Integrated Effects of Digestion Strategy and LC Gradient Architecture Define Host Cell Protein Detectability in CHO Null Harvests

**DOI:** 10.1021/acs.jproteome.6c00097

**Published:** 2026-09-10

**Authors:** Paresh Tank, Fatima D’Souza, Kayalvizhi Nagarajan, Krishnan Muthukalingan, Aruni Wilson Santhosh Kumar, Neelamegam Ramesh Kumar

**Affiliations:** † Amity Institute of Biotechnology (AIB), 584806 Amity University, Mumbai − Pune Expressway, Bhatan, Post-Somathne, Panvel, Mumbai, Maharashtra 410 206, India; ‡ Analytical Chemistry Division of Zelle Biotechnology Research and Analytical Services, Zelle Biotechnology Pvt. Ltd., A-7 M.I.D.C. Mira Industrial Area, Western Express Highway, Mira Road, Thane, Maharashtra 401 104, India; § Regenerative Medicine Laboratory, Department of Zoology, School of Life Sciences, 35480 Periyar University, Salem, Tamil Nadu 636011, India; ∥ Vice Chancellor, 30011 Central University of Tamil Nadu, Thiruvarur, Tamil Nadu 610005, India

**Keywords:** Host cell proteins, mass spectrometry, biopharmaceutical analysis, digestion strategy, chromatographic gradient architecture, QTOF mass spectrometry

## Abstract

Accurate characterization of host cell proteins (HCPs) is critical for biologics development, yet analytical outcomes are strongly influenced by the digestion strategy and chromatographic design. Here, we systematically evaluated digestion parameters, including digestion mode, enzyme ratio, and incubation time, together with LC-MS gradient architecture using a CHO null harvest as a controlled model system to isolate workflow-dependent analytical effects from biological variability. Specifically, the study was designed to determine how these variables jointly influence HCP detection and the physicochemical and biological characteristics of the detected protein populations. By comparing reducing and nonreducing digestion approaches across multiple trypsin ratios and incubation times, we found that under low-enzyme conditions, the primary constraint on HCP recovery is insufficient proteolysis rather than chromatographic undersampling. Reconfiguration of the gradient architecture altered peptide sampling efficiency and MS/MS utilization, enabling controlled comparison of digestion workflows under equivalent acquisition constraints. While higher enzyme ratios increased overall HCP identifications, subcellular localization analysis revealed that low-enzyme native digestion preferentially detected secreted and extracellular proteins with limited access to intracellular species. Extension of native digestion partially restored intracellular protein detection but did not fully recapitulate the coverage achieved by higher enzyme workflows. These findings demonstrate that digestion accessibility and chromatographic spacing impose workflow-dependent effects that shape both the depth and biological origin of detected HCPs, underscoring the need to consider the digestion strategy and chromatographic design together in HCP characterization workflows.

## Graphical abstract

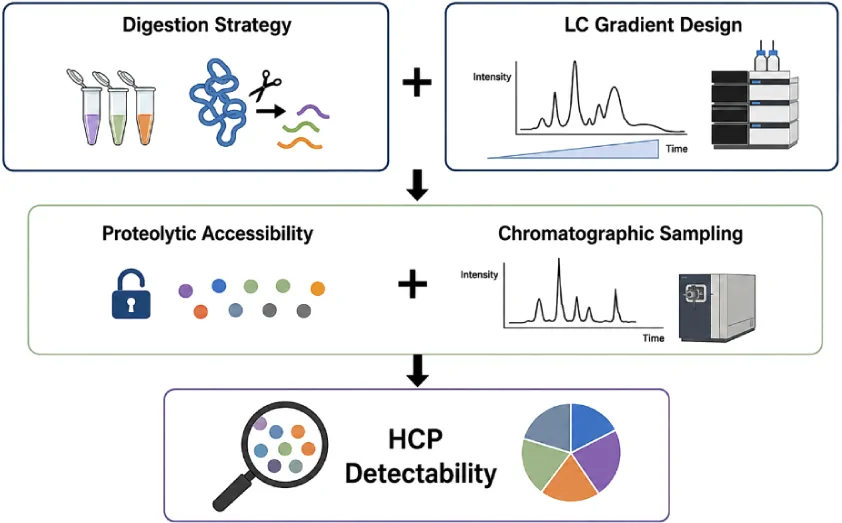

## 1 Introduction

Biopharma production is increasingly dependent on complex expression systems such as CHO, HEK293, and microbial hosts.^1^ These platforms produce a large number of host cell proteins (HCPs), which need to be controlled throughout the entire development process. Even trace amounts may induce immunogenicity, cause degradation of the product, or compromise the stability of the formulation.^2,3^ Several reported cases of product degradation and stability failures associated with residual proteases and lipases highlight the importance of the identification of specific impurities in HCP.^4–9^ Host cell proteins are recognized as critical quality characteristics by the FDA, EMA, WHO, USP, and ICH regulatory frameworks. These frameworks promote detailed molecular characterization rather than relying on the measurement of total HCPs.^10–13^ As the pipeline of biologics expands and biosimilar development accelerates, the need for more in-depth and reproducible monitoring of HCPs is growing.^14^

Process-specific ELISA has long been the main method of quantifying HCP.^15^ ELISAs and related immunoassay platforms are convenient and sensitive, but their analytical performance depends heavily on the antibody coverage and assay design, which may vary depending on the antibody reagents, immunogen, and detection strategy.^16–19^ These restrictions may allow significant impurities to elude detection.^20^ The ELISA also provides only a total concentration and cannot reveal which HCP species persist after purification.^12,21^ This information is increasingly needed for risk assessments, comparative studies, and regulatory submissions at late stage.^22^ LC-MS or MS has been identified as the basic orthogonal method because it can identify and quantify individual proteins in a broad dynamic range.^21,23^ Sensitivity and robustness have improved with advances in sampling techniques, preparation of samples, and bioinformatics.^24–26^ However, the published LC-MS workflows differ considerably in terms of digestion chemistry, enzyme ratios, chromatographic gradients, and platform for the instrumentation. They often focus on only one aspect of the analysis, such as sensitivity or depth, and are rarely assessed under similar conditions. This lack of harmonization limits their practical application in the daily HCP workflow.^27–29^

The main reason for this inconsistency is that digestion chemistry and chromatography design are normally optimized separately.^27,28,30^ Both have a significant effect on the detection of HCPs. The digestion efficiency depends on the folded state of the protein, degree of hydrophobicity, glycosylation, and the proportion of protease to substrate. Native digestion is reported to improve the detection of some low-abundance proteins, but results vary between samples, and there is no clear consensus on when native digestion is superior to traditional reduced peptide mapping.^28,29,31,32^ Chromatographic gradients also vary greatly among studies. The gradient slope and duration have a direct effect on the density of the peptide sample and the number of proteins that can be determined.^23,30,33^ Despite this, few studies have explored how digestion chemistry and gradient structure interact with each other. There is also limited information on how these factors affect the recovery of proteins of high molecular weight, unique peptide formation, or system-adaptive performance.^27,29,34^ These evaluations are essential to build reliable and GMP-relevant HCP characterization.^12^

Another practical challenge is the instrumentation and detection platform, as assay performance can depend on the capabilities and configuration of the analytical system.^35,36^ Many research laboratories still use QTOF-based platforms, although most recent literature on HCP relies on Orbitrap or ion mobility systems.^37–39^ This creates a need for methods that reach high HCP depths in QTOF instruments, using simple digestion settings and chromatography methods that do not require long gradients.^40,41^ These workflows would support the wider adoption of MS for monitoring HCPs in both industrial and academic environments.^42^

The motivation for this study was to determine how the digestion strategy and chromatographic gradient architecture jointly influence the observable HCP population in bottom-up LC-MS analyses. We therefore aimed to delineate how digestion accessibility and chromatographic design constrain the HCP detection depth and the physicochemical and biological characteristics of the detected protein population. To isolate these analytical effects from additional matrix effects introduced by therapeutic proteins and associated depletion strategies, we systematically evaluated the digestion strategy and chromatographic gradient structure using a controlled CHO null-harvest model. We compared reduced peptide mapping and native digestion using trypsin at ratios of 1:20, 1:100, and 1:200. We also compared four LC gradient methods varying in length and slope: an 84 min gradient, a historical 111 min method, a shallow 178 min gradient, and an optimized 112 min gradient. All experiments were performed on the same QTOF platform, and the data were processed consistently. This controlled design enabled a direct comparison of workflow-dependent effects on HCP detection across digestion and chromatographic conditions. The combined data set also enabled assessment of how digestion conditions affect system suitability using a spiked internal standard.

These results provide a practical framework for improving the HCP coverage on QTOF platforms. By linking the digestion chemistry to the chromatographic behavior, the study provides a mechanistic insight into factors that limit or enhance the detection of HCP. It also provides actionable guidance for laboratories that want to set up a robust HCP workflow using MS without relying on special separation techniques or advanced mass spectrometer configurations.

## 2 Materials and Methods

### 2.1 Materials and Reagents

The Rapigest SF surfactant (Waters) was used to denature the digestion. Trypsin Gold (Promega) served as protease in all procedures and was dissolved in 50 mM ammonium bicarbonate directly. Dithiothreitol (DTT), iodoacetamide (IAM), ammonium bicarbonate (ABC), and bovine serum albumin (BS albumin) were bought from Sigma-Aldrich. The Bradford protein test reagent was bought from Bio-Rad. The MassPREP BSA digestion standard (Waters) was used in Experiment 1 to check the suitability of the system. The clarification and buffer exchange were carried out with a centrifugal ultrafiltration device of 3 kDa (Sartorius). Water of LC-MS grade was obtained from J.T. Baker. Acetonitrile and formic acid of LC-MS grade were sourced from Honeywell. The Milli-Q water used for the buffer preparation was produced internally using the Milli-Q Purification System (18.2 MΩ·cm) Merck. Low-binding microcentrifuge tubes and pipet tips (Eppendorf) were used to process the samples, and LC-MS compatible vials (Waters) were used for all injections. Leucine enkephalin (Waters) was used as the lock mass, and sodium iodide (LC-MS grade) was used as the external calibration standard.

### 2.2 CHO Null Harvest Preparation

Day 14 CHO null cell cultures were harvested under feed-batch conditions designed to mimic the feeding regimen and operational strategy of the corresponding production cell line. After harvesting, the culture broth was centrifuged at a rate of 12,000 × g for 10 min to remove the cells and debris. The clarified supernatant was further processed using a 3 kDa centrifugal ultrafiltration device (Sartorius) to remove low-molecular-weight components of the culture medium and concentrate extracellular proteins. In this step, the samples were exchanged with 50 mM ammonium bicarbonate buffer.

The total protein concentration was determined by the Bradford assay. No BSA was added to evaluate the digestion conditions under the legacy gradient, and the clarified harvest was analyzed directly. For the subsequent gradient and digestion assessment, BSA was included as an internal process control for the evaluation of the digestion efficiency and chromatographic performance in the presence and absence of HCP. After quantification of the protein and immediately prior to digestion, BSA was spiked at a concentration of 0.5% (w/w) relative to the total protein mass to ensure that the endogenous proteins and BSA underwent the same digestion conditions. Digests for BSA alone were prepared in parallel by using the same workflow to support the assessment of the suitability of the system.

A single CHO null harvest preparation was used as the common starting material for all digestion and chromatographic workflow evaluations. The objective of the study was to isolate the effects of the digestion strategy and chromatographic gradient architecture on HCP detectability using a constant biological source. Consequently, the reported protein identification counts represent direct workflow outcomes under controlled experimental conditions, rather than statistical estimates derived from replicate analyses.

### 2.3 Digestion Workflows

For the denaturing RPM, samples were prepared in 0.1% of Rapigest SF (Waters), reduced with 10 mM DTT at 60 °C, and alkylated with 20 mM IAM in the dark at room temperature. Then, trypsin was added in a determined ratio and digestion continued for 3 h. After digestion, RapiGest was removed by acidifying the samples with 0.1% of formic acid and incubating for 10 min at 37 °C. For the purposes of native digestion, the samples were treated without surfactants, reductions or alkylations. Trypsin was added directly to the protein solution in the ratio chosen and digestion continued at 37 °C for 3 or 16 h, allowing evaluation of native proteolysis during short and prolonged incubation. No surfactant clearance step was necessary. A modified native procedure (NRPM + R+A) was also used, where samples were first digested under native conditions for 3 h before being exposed to 10 mM DTT and 20 mM IAM under the same conditions as those used for the RPM regimen. This allowed a comparison between proteins that were cleaved natively and those that were made available only after controlled postproteolysis unfolding. After all digestion steps, the peptide solutions were clarified by centrifugation at a rate of 12,000 × g for 5 min, and 10 μg of the resulting peptide mixture was injected for LC-MS/MS analysis.

### 2.4 LC-MS/MS Analysis

All samples were analyzed using a Waters ACQUITY UPLC system coupled to a Xevo G2-XS QTOF mass spectrometer operated in MSE mode. Chromatographic separations were performed on an ACQUITY CSH C18 column (2.1 × 150 mm, Waters) maintained at 65 °C, using mobile phase A (water containing 0.1% formic acid) and mobile phase B (acetonitrile containing 0.1% formic acid) at a flow rate of 0.2 mL min^–1^. Four reversed-phase LC gradients were applied. The 84 min method was initiated at 98% A for 3 min, followed by a linear decrease to 75% A over 45 min and then to 60% A over the subsequent 20 min, before increasing to 2% A and re-equilibrating to 98% A. The historical 111 min gradient, previously reported^42^ was initiated at 98% A for 3 min, followed by a decrease to 85% A over 52 min and then to 65% A over the next 25 min, after which the gradient was increased to 2% A and re-equilibrated to 98% A. The 178 min gradient was initiated at 98% A for 3 min, followed by a decrease to 80% A over 117 min and then to 60% A over the subsequent 40 min, before increasing to 2% A and re-equilibrating to 98% A. The optimized 112 min gradient was initiated at 98% A for 3 min, decreased to 93% A over 5 min and then to 70% A over the next 92 min, followed by an increase to 2% A (100–102 min), an isocratic hold at 2% A (102–105 min) and re-equilibration to 98% A (105–112 min) to complete the run. The QTOF was operated in the ionization mode of the positive electrospray with capillary voltage 2.5 kV, source temperature of 120 °C, desolvation temperature of 350 °C, flow of the ionizing gas at 800 L per hour, and the condensate gas at 50 L per hour. Data from *m*/*z* 50–2500 Da were obtained by using alternating low- and high-energy collision states to generate precursor and fragment ion mass spectra. Leucine enkephalin (*m*/*z*. 556.2766) was used as reference mass, and sodium iodide was used as external calibration. All digests, including BSA-only controls, were analyzed under identical LC-MS conditions to allow direct comparison across digestion workflows and chromatographic gradients.

### 2.5 Protein Identification and Data Processing

Raw Waters MS^E^ data were imported into Protein Metrics Byos (Protein Metrics, CA) through the vendor-supported Waters data interface for peptide and protein identification. Chromatographic overlays and visualization of LC-MS data were generated using Waters Connect (Waters Corporation). Database searching and protein assignment were performed within the Byos environment by using workflows compatible with Waters MS^E^ acquisition data. Protein sequences related to *Cricetulus griseus* (organism ID: 10029) were obtained from UniProt, consisting of 83 428 protein sequences (downloaded September 2025). Database searches were performed with trypsin specificity, allowing for up to 2 missed fragments. Carbamidomethylation of cysteine was used as a fixed modification for the reduced and alkylated samples, while methionine oxidation and asparagine-glutamine degradation were included as common variable modifications. A contaminant protein library and automatically generated decoy sequences were included to support the scoring and validation. Common laboratory contaminants, including keratins and other proteins contained within the contaminant library, together with digestion-related artifacts such as trypsin autolysis products, were excluded from the reported HCP counts.

Peptide spectrum matches were accepted with a 2% false discovery rate (FDR) using Byonic’s autodecoy workflow with a 50% reverse-count setting. After applying FDR, the additional filter retained only peptides with a mass error between −10 and +10 ppm, a minimum weight of peptides of 350 Da, and a Byonic score of 100. These criteria were applied to support high-confidence peptide assignments for low abundance HCP characterization. A protein was accepted if supported by at least one unique peptide, defined as a peptide sequence mapping exclusively to a single protein (“1 in 1”). From Byos, processed peptide and protein tables were exported for further analysis, including MW-PI mapping, chromatographic comparison, and evaluation of digestion pathways.

### 2.6 System Suitability Evaluation

The suitability of the system was monitored by means of quality control samples such as BSA, processed and analyzed together with all study procedures. The suitability assessment focused on the chromatographic stability and performance indicators, including the number of detectable BSA peptides, the extracted ion chromatogram (XIC) peak quality, and the apex retention time. In the digestion optimization experiments, matrix-spiked BSA was also used to explain the digestion-dependent differences in peptide generation between the workflows. These metrics were used to verify that the performance of the LC-MS remained consistent between sample sets and analysis runs. For the relative retention time (RRT) analysis in the digestion optimization experiments, the reference peptide was selected as a high-intensity BSA peptide eluted in the central region of the chromatographic gradient. Peptide HLVDEPQNLIK has been used to standardize RRT normalization across injections. Peptides detected in only one of the two bracketed SST injections or showing a low signal intensity were excluded from the RRT-based assessment.

## 3 Results

### 3.1 Impact of Digestion Chemistry on HCP Recovery Using the Legacy LC Gradient

Figure 1A shows the chromatographic behavior of the four digestion conditions with the historical gradient of 111 min. RPM digestion produced dense, richly structured BPI chromatograms with a number of different peptide signals distributed over the gradient, whereas NRPM digestion produced visibly simpler traces. The decrease in the ratio of trypsin from 1:20 to 1:200 had little effect on the complexity of the RPM chromatogram, but further reduced the peak density of NRPM traces, suggesting that native digestion produced fewer peptides because of the reduced availability of protease. Bracketed injections of a manufacturer-digested MassPREP BSA standard showed stable chromatographic and MS performance across the batch. Complete lists of protein identifiers for all digestion steps and chromatographic gradients are given in Supplementary tables S1 – S12.

**Figure 1:**
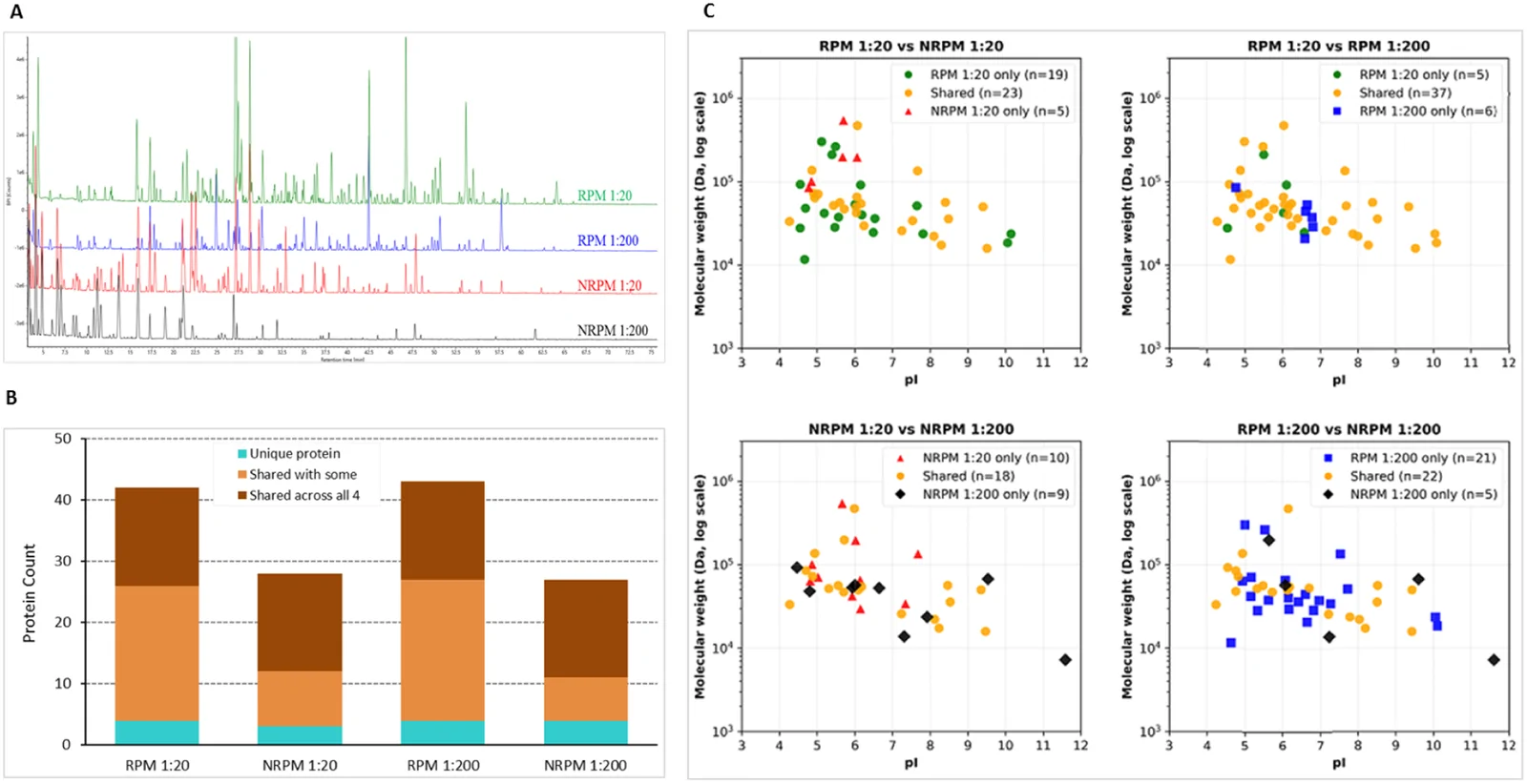
Digestion-dependent differences in chromatographic profiles, protein identifications, and physicochemical distributions under the historical 111 min LC gradient. (A) Overlaid base peak intensity (BPI) chromatograms for the four digestion conditions (RPM 1:20, RPM 1:200, NRPM 1:20, and NRPM 1:200). RPM workflows produced more complex and feature-rich chromatographic profiles, whereas NRPM traces were visibly simpler and showed further reduction in peak density at the lower trypsin ratio. (B) Protein identification counts for each digestion condition, grouped into proteins detected uniquely in that condition, proteins shared with some conditions, and proteins shared across all four. RPM yielded the highest and most consistent depth across enzyme ratios, while NRPM exhibited reduced coverage, particularly at 1:200. (C) Molecular weight-pI distributions for proteins identified in each pairwise comparison of digestion conditions (RPM 1:20 vs NRPM 1:20; RPM 1:20 vs RPM 1:200; NRPM 1:20 vs NRPM 1:200; and RPM 1:200 vs NRPM 1:200). In each comparison, proteins are classified as unique to one workflow or shared between both. Shared proteins occupied a broad MW-pI space, RPM-unique proteins showed wide physicochemical diversity, and NRPM-unique proteins were fewer and displayed more restricted distributions, especially at the lower enzyme ratio.

These differences were reflected in the identification profiles of proteins as shown in Figure 1B. The RPM 1:20 and RPM 1:200 methods yielded the highest total protein content, each with a significant contribution from unique proteins and from proteins that are common to some but not all conditions. Conversely, NRPM 1:20 and NRPM 1:200 showed a decrease in total coverage, mainly due to a decrease in the shared and unique protein categories. The lowest profile was observed in NRPM 1:200, which showed a loss of both unique identifiers and proteins that are shared with other workflows. Across enzyme ratios, RPM maintained a wide and stable distribution of unique and partially common proteins, whereas NRPM showed a clear decrease in coverage at the lower levels of trypsin. A consistent protein sequence was shared across all four processes, suggesting that the core CHO HCP population is reliably recovered, regardless of the digestion chemistry.

The molecular weight (MW) and isoelectric point (pI) distributions of proteins identified across digestion contrasts are shown in Figure 1C. A comparison of RPM 1:20 and NRPM 1:20 yielded 47 proteins in total, comprising 19 proteins unique to RPM 1:20, 5 unique to NRPM 1:20, and 23 shared between the two workflows. While shared proteins spanned a broad MW-pI space, NRPM-specific identifications were confined to a narrower physicochemical range, indicating reduced proteolytic accessibility under native conditions, despite higher enzyme availability.

Reducing the enzyme ratio within denaturing digestion had minimal impact on physicochemical coverage. A comparison of RPM 1:20 and RPM 1:200 identified 48 proteins, with 37 shared between conditions and only a small number of unique identifications for each workflow (5 for RPM 1:20 and 6 for RPM 1:200). The strong overlap and comparable MW–pI distributions indicate that denaturing digestion is robust to enzyme limitation within the range evaluated.

A comparison of NRPM 1:20 and NRPM 1:200 identified 37 proteins in total, with 18 proteins shared between the two conditions and comparable numbers of unique identifications for each workflow (10 for NRPM 1:20 and 9 for NRPM 1:200). The overall similarity in protein counts indicates that, within native digestion, reducing the enzyme ratio has only a modest effect on the number of detectable HCPs. However, across both NRPM conditions, unique proteins remained confined to a restricted MW–pI space, consistent with limited proteolytic accessibility under native conditions.

This effect was further accentuated when comparing RPM 1:200 and NRPM 1:200. Of the 48 proteins identified, 22 were shared, while RPM 1:200 yielded 21 unique proteins compared to only 5 unique proteins for NRPM 1:200. RPM-specific proteins covered a substantially broader MW-pI range, whereas NRPM-specific identifications remained confined, highlighting the combined impact of the enzyme limitation and native protein conformation.

Collectively, these data demonstrate that digestion chemistry is the dominant determinant of HCP physicochemical coverage under the historical 111 min gradient. Denaturing digestion consistently enables broader and more uniform access to the HCP proteome, whereas native digestion restricts detectable proteins to a narrower subset defined by proteolytic accessibility, with the enzyme ratio exerting only a modest secondary effect.

### 3.2 Impact of Chromatographic Gradient Architecture on HCP Recovery

The effect of the chromatographic design on the detection of HCP was assessed by using the four LC gradients shown in Figure 2. The 84-, 111-, 178-, and 112 min gradients each resulted in different elution patterns (Figure 2A). The 84 min method compressed the majority of peptide features into the early part of the run, resulting in extensive coelution and a reduction in the efficiency of the MS and MS sampling. The 111 min method reduced some of this compression but still showed peak clustering in the first half of the gradient. The 178 min shallow gradient distributed the features more evenly over the chromatographic window, but its low gradient resulted in wider peaks with lower peak intensity. In contrast , the 112 min optimized method resulted in a more even dispersion of chromatographic characteristics and improved resolution in the intermediate gradient region, where many of the low-level HCP peptides elute.

**Figure 2:**
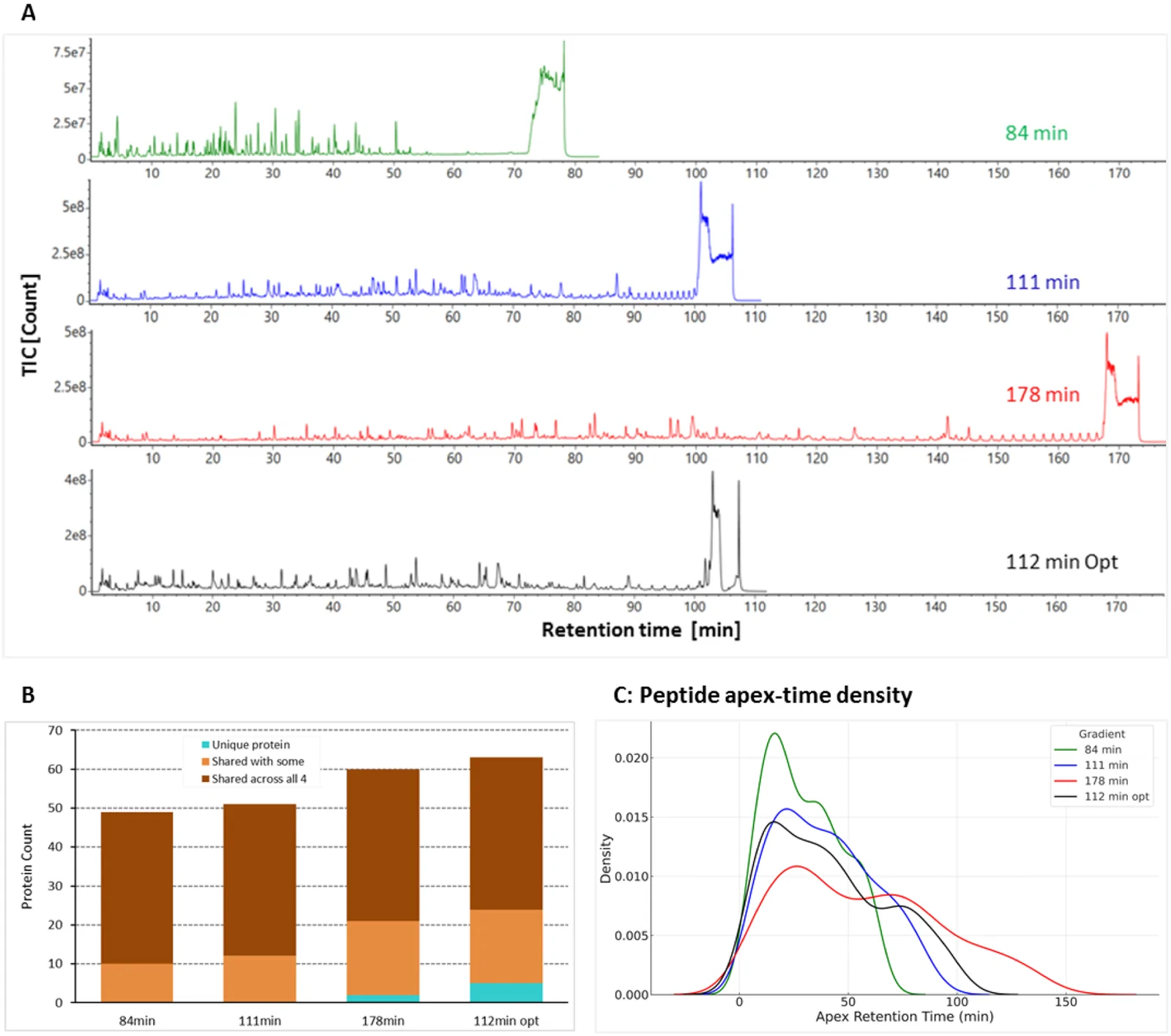
Influence of chromatographic gradient architecture on HCP detection depth. (A) Total ion chromatograms for the 84-, 111-, 178-, and optimized 112 min gradients. The 84 min method shows compressed, highly coeluting features, whereas the 111- and 178 min gradients spread features more broadly but inconsistently. The optimized 112 min gradient provides more uniform peak spacing and improved midelution resolution, enhancing sampling of low-abundance HCP peptides. (B) Protein identifications across the four gradients. The optimized 112 min method yields the highest number of unique proteins and the deepest overall HCP coverage. (C) Kernel-density distributions of peptide apex retention times. The 84 and 111 min gradients exhibit early elution clustering, while the 178 min method shows broad but sparsely sampled dispersion. The 112 min gradient achieves a balanced apex-time profile, reflecting improved chromatographic separation and MS/MS utilization.

These chromatographic properties were directly translated into differences in the depth of protein detection (Figure 2B). Legacy gradients identified 54 (84 min), 56 (111 min), and 65 (178 min) proteins, while the optimized 112 min method yielded 69 proteins, the highest of any of the conditions. The increase in identification by the 112 min method was due to increases in both unique proteins and proteins shared with a subset of other gradients, suggesting that the increased range of expression was due to a more efficient use of MS rather than a simple redistribution of the same peptides. Although the 178 min gradient yielded the highest total number of detected peptides, this did not translate to a proportional increase in protein identifications. This observation likely reflects broader chromatographic peak widths and reduced effective MS/MS sampling density under shallow gradient conditions.

The distribution of the exposure retention time further highlighted the different sampling characteristics of the different gradients (Figure 2C). The 84- and 111 min methods showed sharp peaks in early elution density, reflecting coelution and limited MS and MS/MS possibilities. The 178 min gradient showed the widest distribution but a lower overall density, indicating an inefficient duty cycle coupling despite prolonged separation. The optimized 112 min method showed a more balanced density profile, with a higher peak sampling density along the middle of the gradient. This distribution is consistent with the improved detection performance and indicates that the optimized gradient achieves a better balance between the chromatographic resolution and MS acquisition bandwidth.

The suitability of the system based on the unique peptide recovery of the BSA is shown in Supplementary Figure S1. Using the same aliquot containing BSA for all injections, the 84 min gradient produced 33 unique peptides of BSA, while the 111 min, 178 min, and optimized 112 min gradients produced 40, 41, and 40 unique peptides of BSA, respectively. This pattern shows a clear improvement in the efficiency of peptide sampling when switching from the 84 min compressed gradient method to a longer or reformed gradient. Importantly, the three extended gradient methods produced almost identical BSA results, which indicates that the differences in the detection of HCP between these methods are due to the chromatographic characteristics of the sample and not to the variability of sample handling. These observations support that the increased HCP depth achieved by the optimized 112 min gradient reflects an improved use of the MS acquisition bandwidth rather than differences in the availability of the analytes.

Combined, the data in Figure 2 show that the chromatographic gradient structure has a significant effect on the detection of HCP regardless of the digestion chemistry. The optimized 112 min method improves both the sample-level and the protein-level coverage compared to the legacy methods and outperforms even the extended 178 min gradient, despite the shorter running time. These findings indicate that the 112 min gradient represents a more efficient analytical configuration for deep HCP profiling and provide a benchmark for the integration of digestion and chromatographic strategies in subsequent workflow optimization.

### 3.3 Impact of Digestion Time and Enzyme Ratio on Physicochemical Coverage of Native Workflows

Four digestion strategies evaluated under the LC-optimized 112 min LC method revealed clear differences in the extent of HCP detection and in the physicochemical profiles (Figure 3). RPM 1:20 (3 h) and NRPM 1:20 (3 h R+A) produced the deepest profiles, with a total of 68 and 75 proteins, respectively, while NRPM 1:100 (3 h) yielded only 17 proteins and NRPM 1:100 (16 h) recovered an intermediate 46 proteins (Figure 3B). These trends are consistent with the chromatographic complexity seen in Figure 3A, where the RPM and NRPM 1:20 R+A traces exhibit dense feature distributions relative to the sparse profiles of the low-trypsin NRPM workflows.

**Figure 3:**
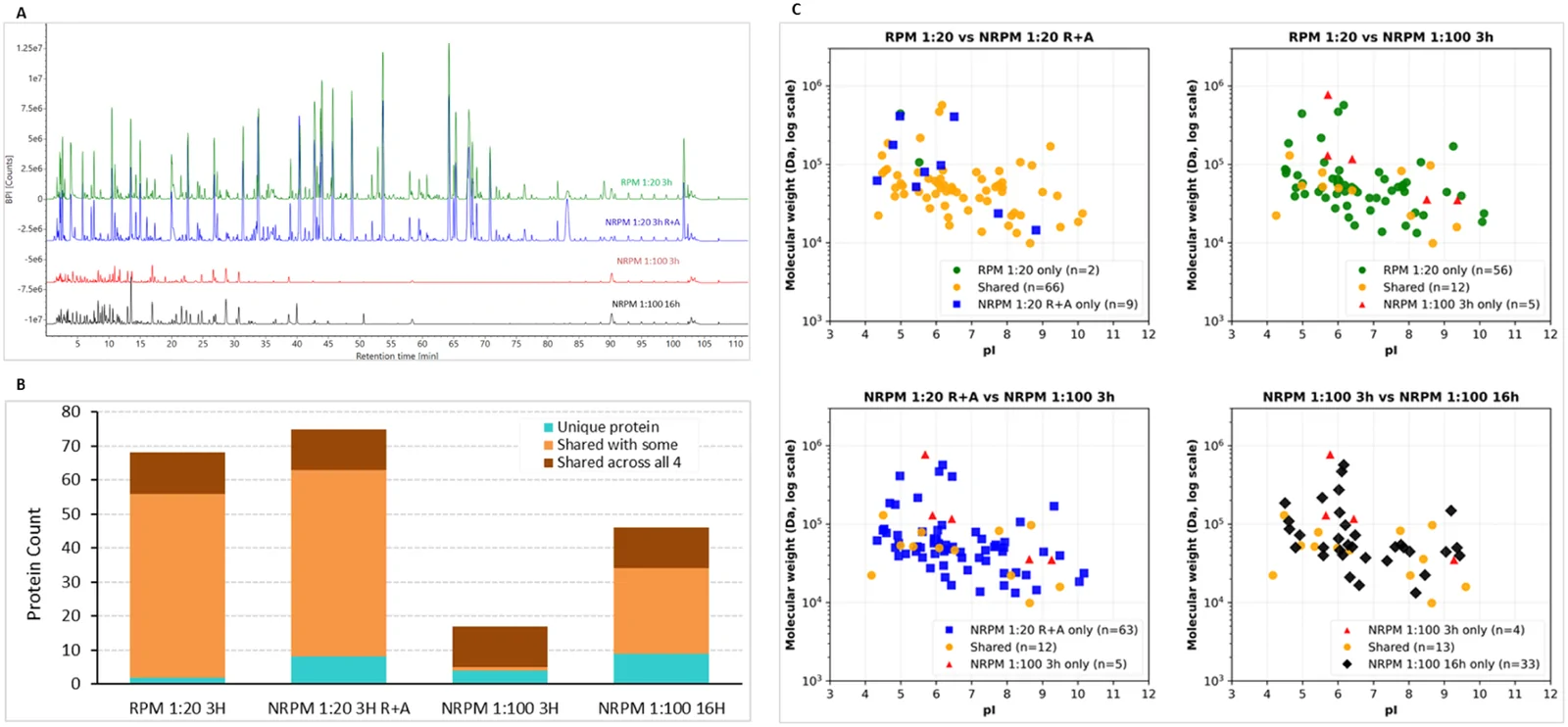
Impact of digestion mode, enzyme ratio, and incubation time on chromatographic profiles, protein identifications, and MW–pI distributions under the optimized 112 min LC gradient. (A) Overlaid BPI chromatograms for RPM 1:20 (3 h), NRPM 1:20 (3 h R+A), NRPM 1:100 (3 h), and NRPM 1:100 (16 h). RPM and NRPM 1:20 R+A exhibited dense peptide feature profiles, whereas NRPM 1:100 (3 h) produced sparse MS responses that improved upon extending digestion to 16 h. (B) Protein identification counts categorized as unique, shared with some workflows, or shared across all four conditions. RPM 1:20 and NRPM 1:20 R+A achieved the greatest depth, NRPM 1:100 (3 h) yielded the lowest coverage, and prolonging the low-enzyme digestion to 16 h partially restored identifications. (C) MW–pI distributions for the four pairwise workflow comparisons. High-enzyme workflows (RPM 1:20 and NRPM 1:20 R+A) recovered a broad physicochemical space, whereas low-enzyme native digestion (1:100, 3 h) displayed restricted coverage that expanded with extended incubation. Overall, both trypsin availability and digestion time jointly shaped the accessible HCP physicochemical landscape.

The MW-PI pairwise comparison provides further mechanistic insight into how the enzyme ratio and the digestion time shape the available HCP space (Figure 3C). In comparison to RPM 1:20 (3 h) and NRPM 1:20 (3 h R+A), 66 proteins were pooled, with only a small number of proteins being unique to each of the two pathways (2 unique for RPM; 9 unique for NRPM R+A). These proteins were found in comparable MW-PI ranges, suggesting that if the availability of trypsin is sufficient, short-term digestion yields a broadly similar physicochemical profile, irrespective of whether denaturation occurs prior to or following proteolysis. Notably, several unique NRPM proteins were distributed in regions with a higher molecular weight (>100 kDa), suggesting that unfolding after native digestion may still release otherwise inaccessible peptides for MS and MS/MS sampling.

The difference was more pronounced when comparing NRPM 1:20 (3 h R+A) with NRPM 1:100 (3 h). The reduction in the trypsin ratio from 1:20 to 1:100 in the native state reduced the available HCP space, resulting in 63 proteins unique for the R+A 1:20 process and only 5 proteins unique for the 1:100 digestion (3 h) process, with a total of 12 proteins shared. Shared proteins clustered in the restricted MW-PI region, consistent with incomplete cleavage under enzyme-depleted conditions. This pattern is consistent with the observations in Section 2.1 where low protease availability reduced NRPM performance even in a long historical range.

The extension of the digestion period at a low trypsin ratio (NRPM 1:100, 16 h) partially restored the recovery of the HCP. Compared to 1:100 (3 h), the 16-h workflow increased total identification from 17 to 46 and significantly increased the MW-PI coverage. MW-PI showed 33 proteins unique to prolonged incubation, 4 unique to 3-h digestion, and 13 common proteins. These additional identifications covered a wider range of acid to neutral pI values and included several proteins in the mid-MW range that are absent in the shorter digest. This shows that under native conditions, long-term digestion may compensate for the limited access to protease but not to the extent that higher-enzyme workflows can.

A comparison of NRPM 1:20 (3 h R+A) and NRPM 1:100 (16 h) shows that NRPM is the practical upper limit of native digestion rescue. Despite the significant improvement at 16 h, the R+A workflow with higher enzyme levels still provided a wider physicochemical coverage, yielding 39 unique proteins compared to 10 unique proteins in the 16-h digest with low enzyme levels, with 36 proteins shared between the two. NRPM 1:20 R+A unique proteins occupied a broader MW-PI region, confirming that controlled denaturation, whether applied prior to or following proteolysis, is still necessary for a deeper recovery of HCP.

To further elucidate the differences in HCP recovery dependent on digestion, we examined the subcellular origin of the proteins determined by the LC-MS optimized 112 min method (Figure 4). The proteins detected in high-enzyme workflows (RPM 1:20 and NRPM 1:20 R+A) included a wide range of intracellular compartments, including cytosolic, membrane-bound, ER, and Golgi-bound proteins, in addition to the species that are excreted. In contrast, native digestion of low-enzyme content at 1:100 (3 h) resulted in a significantly reduced profile dominated by secreted and extracellular proteins, with minimal intracellular protein representation. Extending native digestion to 16 h partially restored intracellular identification, suggesting that prolonged incubation may improve access to structurally restricted proteins, although coverage remains lower than that obtained with higher enzyme-mediated processes. In line with these trends, several proteins with functions previously associated with HCP risk were detected predominantly under higher-enzyme or extended-digestion conditions (Table 1), following the same digestion-dependent accessibility patterns observed at the global proteome level.

**Figure 4:**
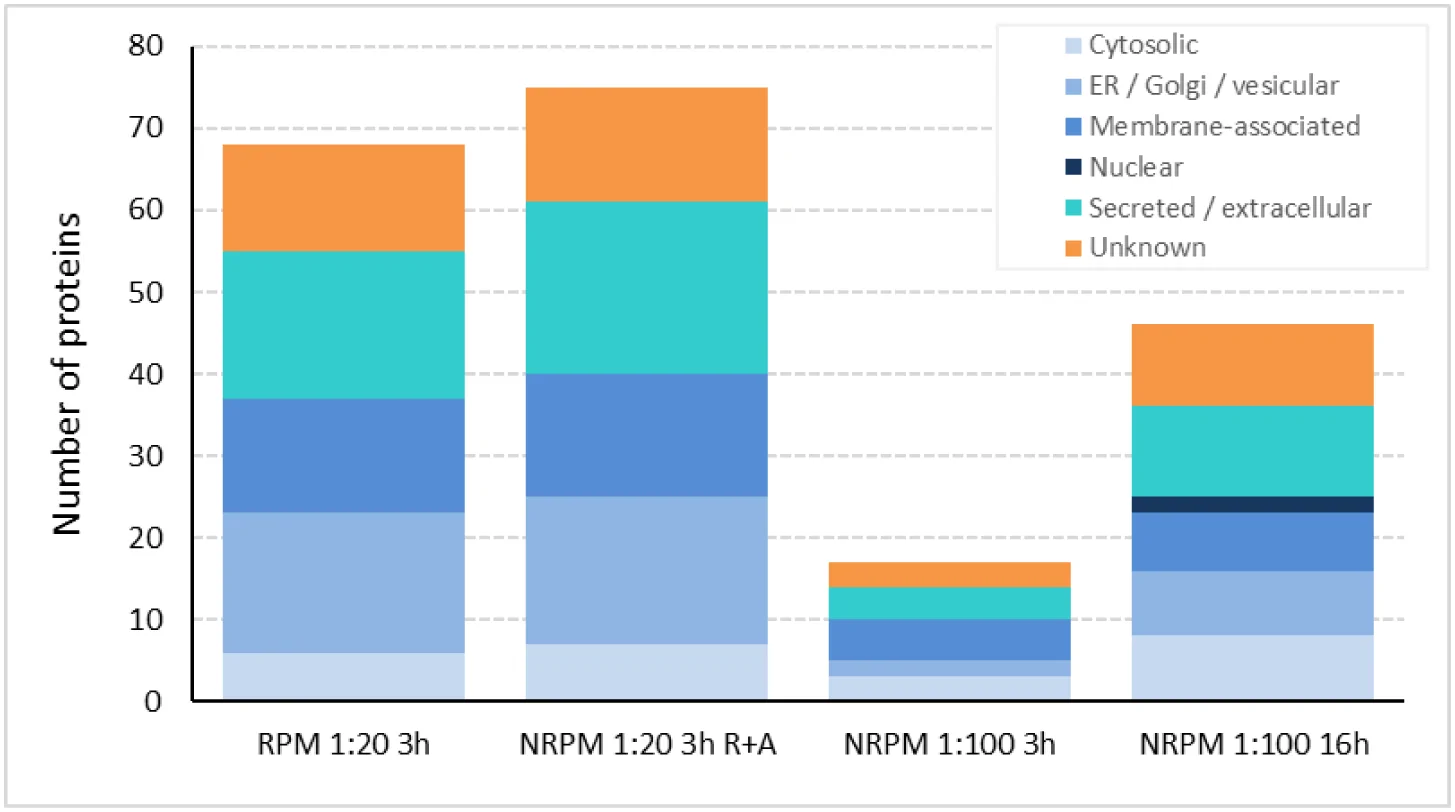
Digestion-dependent subcellular localization bias of detected host cell proteins under optimized LC–MS conditions. Stacked bar plots show the number of host cell proteins (HCPs) identified under each digestion workflow, grouped by UniProt subcellular localization annotations. Proteins annotated as cytosolic, nuclear, membrane-associated, or localized to the ER/Golgi/vesicular system are shown as subcategories within a single intracellular-associated block, while secreted/extracellular and unannotated proteins are shown separately. High-enzyme workflows (RPM 1:20 and NRPM 1:20 R+A) recovered a broad spectrum of intracellular and extracellular protein classes, whereas low-enzyme native digestion at 1:100 (3 h) was dominated by secreted/extracellular proteins. Extension of native digestion to 16 h partially restored detection of intracellular-associated proteins, indicating that digestion accessibility influences the apparent subcellular origin of detected HCPs (Supplementary Table S16).

**Table 1:**
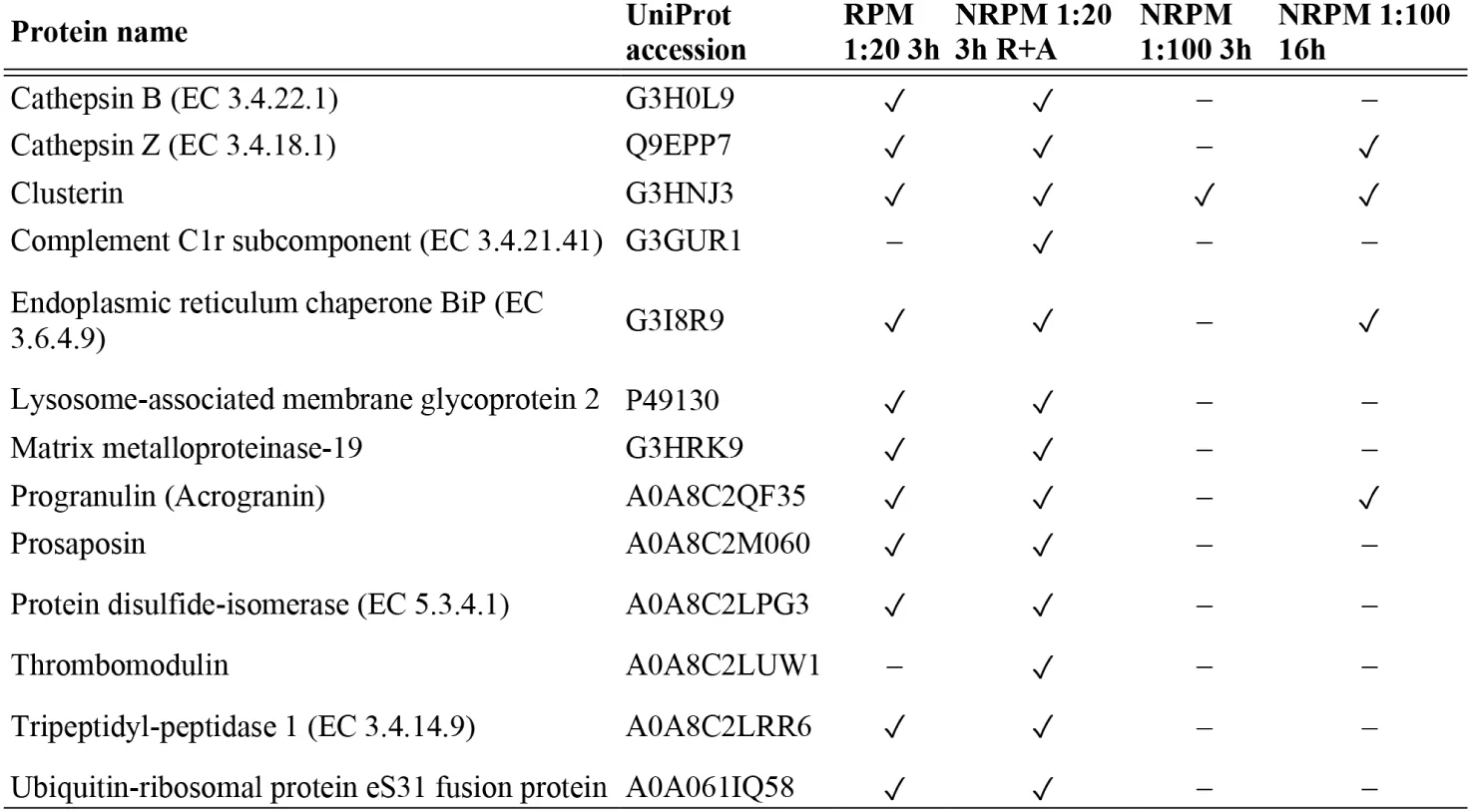
Detection of Selected High-Risk Functional Host Cell Proteins across Digestion Workflows

Bracketed system suitability injections acquired under the optimized 112 min LC method showed a high degree of consistency in the relative retention time of peptides between injections (Figure 5A). The extraction-ion chromatogram (XIC) ratios of peptide levels between the bracketed injections were close to unity with a narrow distribution (Figure 5B), which confirmed the stable chromatographic and MS performance across the analytical batch.

**Figure 5:**
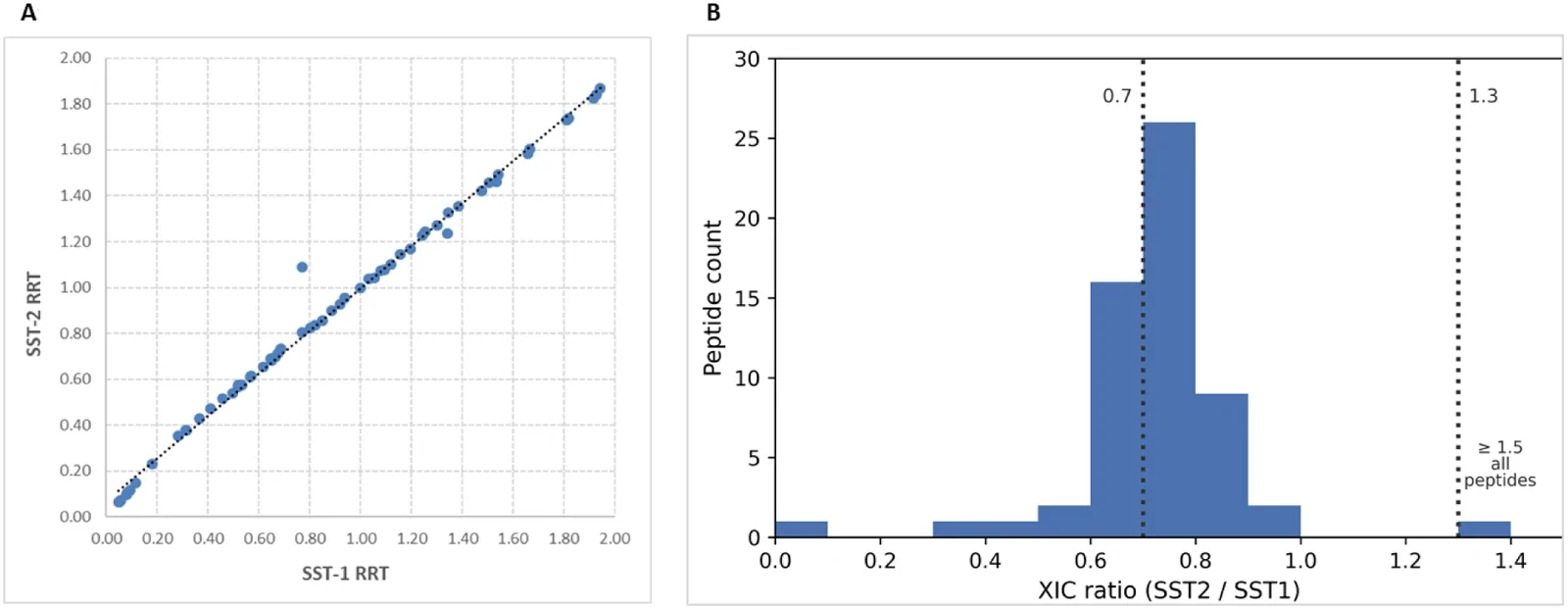
Bracketed system suitability performance under the optimized 112 min LC method. (A) Relative retention time (RRT) correlation between two bracketed SST injections acquired under identical LC–MS conditions, demonstrating chromatographic stability across the batch. (B) Distribution of peptide-level extracted-ion chromatogram (XIC) ratios (SST-2/SST-1) for bracketed SST injections. Dashed vertical lines indicate reference boundaries used to visualize peptide-level signal variability under stable LC–MS conditions.

The matrix-spiked BSA controls reflected differences in peptide recovery dependent on digestion (Supplementary Figure S2). The 1:20 (3 h) and NRPM 1:20 (3 h R+A) yielded 38 and 43 unique BSA peptides, respectively. In contrast, NRPM 1:100 (3 h) produced no detectable BSA peptides, and a prolonged digestion time of 16 h yielded one unique peptide. A comparison of the SST and spiked SST peptide detection rates for different digestion workflows showed that high-enzyme conditions preserved the qualitative recovery of peptides, while low-enzyme native digestion resulted in a significant loss of detectable SST peptides (Supplementary Table S14). The distribution of XIC at the peptide level in spiked SST samples varied between digestion conditions in comparison to bracketed SST injections, reflecting the additional matrix and digestion-dependent effects on signal intensity (Supplementary Table S15).

Together, these results indicate that the native digestion rate is dependent on the availability of trypsin and incubation time. Short native digestion with low enzyme ratios severely limits the physicochemical diversity, while prolonged digestion partially restores access but does not fully restore the coverage of denatured workflows. These findings further highlight the need for co-optimization of the digestion strategy with chromatographic resolution in order to achieve a reliable identification of HCP on QTOF platforms.

## 4 Discussion

Mass spectrometry is now the primary tool for monitoring host cell proteins in the development of bioprocesses.^16,21^ Its use has increased due to improvements in sample preparation, liquid chromatography, and data-collection strategies.^27^ Many studies have reported improved recovery of HCP using approaches such as native digestion, SP3 processing, or multidimensional LC.^28,31,43–46^ However, these methods are often optimized for a particular type of sample or instrument. Therefore, their reported performance does not demonstrate how digestion chemistry and chromatography work together in the single-dimensional LC-MS workflow used by most development laboratories. Our study addresses this need by examining the digestion accessibility and chromatographic resolving power within a common analytical framework.

In all experiments, the digestion chemistry had the greatest effect on the detection of the HCP. Denaturing reduced peptide mapping produced a broad protein coverage and a wide range of MW and pI values. This pattern reflects a more complete proteolysis and efficient peptide sampling. In contrast, native digestion with a reduced trypsin ratio resulted in a substantially lower number of protein identifications. This behavior is consistent with the limited cleavage of the folded substrates in the presence of low enzyme availability. Although some reports suggest that native digestion may improve detection in specific cases,^28,31^ our results indicate that its efficacy depends strongly on trypsin availability and the degree of protein unfolding achieved during digestion. The introduction of reduction and alkylation after native digestion increased the level of detection of higher-molecular-weight and structurally complex proteins. This finding suggests that unfolding, whether pre- or postproteolysis, remains necessary for a wide coverage of HCP.

The chromatographic gradient design also had a clear influence on the efficiency of peptide sampling. The optimized gradient of 112 min increased the detection depth compared with the legacy gradient and the extended method of 178 min. This improvement was due to the fact that the optimized gradient distributed peptides more evenly and increased the peak density in the midelution area. Recent studies have shown that LC architecture is important for sampling low-abundance peptides,^29,30^ and our results support this view. Gradients that produce clustered or sparsely distributed features give less identification, even if the digestion chemistry is favorable. This indicates that the LC design plays a direct role in the conversion of available peptides to detectable proteins.

From a practical perspective, each workflow presents distinct advantages and limitations. Denaturing digestion combined with higher trypsin availability provided the broadest HCP coverage and most consistent recovery of intracellular proteins but required additional sample-processing steps and reagent handling. Native digestion offered a simplified workflow and preferential recovery of readily accessible extracellular proteins, although the coverage became increasingly dependent on enzyme availability and digestion time. Similarly, extended chromatographic gradients increased separation space but required greater instrument occupancy, whereas optimized gradient architectures sought to balance the analytical depth with throughput. These findings indicate that no single workflow is universally optimal and that digestion strategy and chromatographic design should be selected according to the analytical objective, whether prioritizing maximum HCP coverage, workflow simplicity, or operational efficiency.

When the digestion and chromatographic effects were assessed together, clear interdependence was observed. The workflows that produced more peptides during the digestion phase benefited most from the improved chromatography spacing. On the other hand, native digestion with reduced enzyme was not significantly improved at any gradient. Extending native digestion at a 1:100 ratio from 3 to 16 h increased protein identification, suggesting that prolonged incubation would progressively increase access to structurally restricted cleavage sites. However, even extended native digestion did not achieve the coverage obtained by denaturing or R+A-assisted digestion. This finding indicates that, under conditions of limited enzyme availability, proteolytic accessibility represents the dominant constraint on HCP detection rather than chromatographic undersampling and that this constraint introduces a bias toward extracellular protein detection.

The measurements of the system suitability were consistent with these mechanistic observations. RPM 1:20 and NRPM 1:20 with reduction and alkylation resulted in strong and reproducible signals of BSA, while native digestion with a low-enzyme content resulted in minimal signals of BSA even after prolonged incubation. This supports the conclusion that the differences in HCP recovery are mainly due to proteolytic activity and not to variations in LC-MS performance.

The number of HCPs detected in this study reflects both the biological composition of the clarified CHO null harvest material and the analytical workflow applied. Clarified harvests are expected to contain predominantly extracellular and secreted proteins, together with a limited contribution from intracellular species released during normal culture operations. Contemporary LC-MS/MS studies have demonstrated that sample preparation strategy, digestion accessibility, chromatographic design, and acquisition methodology all influence the depth of detectable HCP coverage.^28,29,37,40,43,44^ In this context, the identification differences observed here are most appropriately interpreted as workflow-dependent effects on protein accessibility and peptide sampling efficiency rather than as measures of absolute proteome completeness. The results therefore provide insight into how the analytical workflow design influences the observable HCP population within a controlled harvest matrix. Accordingly, the workflow-dependent differences reported here should be interpreted as comparative analytical outcomes obtained under a consistent identification framework rather than as statistically validated differences between workflows.

Together, these results provide a mechanistic framework for understanding HCP detectability on QTOF-based LC-MS platforms. By considering digestion chemistry and chromatographic structure as interdependent analytical variables, the study clarifies how workflow design constrains protein accessibility and sampling efficiency without the reliance on specialized instrumentation or complex separation schemes. This Perspective complements ongoing advances in sample preparation and acquisition strategies and offers practical guidance for establishing reproducible LC-MS-based HCP characterization workflows in bioprocess development.

## 5 Conclusion

This study demonstrates that digestion chemistry and chromatographic design act as interdependent analytical variables that jointly determine the depth and consistency of HCP detectability on QTOF-based LC-MS platforms. Denaturing digestion provided more consistent proteolytic accessibility, whereas native digestion required sufficient enzyme availability and controlled unfolding to expand coverage beyond predominantly extracellular species. Modulation of the gradient architecture influenced peptide sampling efficiency and MS/MS utilization, enabling controlled comparison of digestion workflows under comparable acquisition constraints. Within the biological constraints of clarified harvest materials, these workflows provided access to a substantial portion of the detectable extracellular proteome, illustrating how analytical design choices shape the observable HCP space.

A limitation of the present study is that evaluations were performed using a single CHO null-harvest preparation without replicate LC-MS analyses and without the inclusion of a therapeutic product matrix. Consequently, the conclusions are restricted to a comparative assessment of workflow-dependent analytical effects within a controlled CHO null-harvest model and should not be interpreted as representing the complete analytical workflow used for therapeutic product characterization or as a definitive inventory of CHO HCPs. Future work should extend this framework to product-containing matrices, additional harvest conditions, replicate LC-MS analyses, and evaluations under alternative protein identification criteria, including protein-level false discovery rate and unique peptide requirements. These studies will further clarify the general applicability of the observed workflow-dependent effects and strengthen our confidence in the robustness of the observed trends.

Looking forward, broader adoption of MS-based HCP characterization will benefit from continued efforts to simplify and harmonize sample preparation practices, develop system-suitability and calibration tools appropriate for QTOF platforms, and establish shared reference materials to facilitate interlaboratory comparison.^47^ Extension of this framework to additional harvest types, upstream conditions, and downstream processing contexts will further clarify how workflow-dependent constraints influence HCP detectability across bioprocesses. Together, these advances will support more consistent and interpretable application of LC-MS-based HCP analysis as a practical tool in bioprocess development.

## Supplementary Material





## Data Availability

The mass spectrometry proteomics data have been deposited to the ProteomeXchange Consortium via the PRIDE partner repository with the data set identifier PXD075008.

## Notes

Figure S1. Chromatographic gradient–dependent peptide sampling assessed using BSA controls. Figure S2. Digestion-dependent system suitability performance under the optimized 112 min LC method (PDF)
Table S1 (XLSX). Historical 111 min gradient, RPM digestion, trypsin 1:20, 4 h Table S2 (XLSX). Historical 111 min gradient, NRPM digestion, trypsin 1:20, 4 h Table S3 (XLSX). Historical 111 min gradient, RPM digestion, trypsin 1:200, 4 h Table S4 (XLSX). Historical 111 min gradient, NRPM digestion, trypsin 1:200, 4 h Table S5 (XLSX). Gradient 84 min, RPM digestion, trypsin 1:20, 3 h Table S6 (XLSX). Gradient 111 min, RPM digestion, trypsin 1:20, 3 h Table S7 (XLSX). Gradient 178 min, RPM digestion, trypsin 1:20, 3 h Table S8 (XLSX). Gradient 112 min, RPM digestion, trypsin 1:20, 3 h Table S9 (XLSX). Digestion 112 min gradient, RPM digestion, trypsin 1:20, 3 h Table S10 (XLSX). Digestion 112 min gradient, NRPM digestion, trypsin 1:20, 3 h, R+A Table S11 (XLSX). Digestion 112 min gradient, NRPM digestion, trypsin 1:100, 3 h Table S12 (XLSX). Digestion 112 min gradient, NRPM digestion, trypsin 1:100, 16 h Table S13 (XLSX). Peptide-level retention time and signal metrics for bracketed SST injections Table S14 (XLSX). Comparison of peptide detectability between bracketed SST and matrix-spiked SST Table S15 (XLSX). Peptide-level XIC ratio distributions for bracketed and matrix-spiked SST Table S16 (XLSX) (XLSX)
