# Supplementary material for: Integrated Effects of Digestion Strategy and LC Gradient Architecture Define Host Cell Protein Detectability in CHO Null Harvests
Source: J Proteome Res. 2026 Sep 10;25(10):5200–11. doi: 10.1021/acs.jproteome.6c00097 (PMC13640511; doi:10.1021/acs.jproteome.6c00097)
Supplement: Supplementary file 1 [file pr6c00097_si_001.pdf]

Supporting Information

Integrated effects of digestion strategy and LC gradient architecture define host cell protein detectability in CHO null harvests

Paresh Tank[a,b], Fatima D'Souza[b], Kayalvizhi Nagarajan[c], Krishnan Muthukalingan[d], Aruni Wilson Santhosh Kumar[a], Neelamegam Ramesh Kumar[a*]

[a]Amity Institute of Biotechnology (AIB), Amity University, Mumbai – Pune Expressway, Bhatan, Post-Somathne, Panvel, Mumbai. Maharashtra – 410 206. INDIA.

[b]Analytical Chemistry Division of Zelle Biotechnology Research and Analytical Services, Zelle Biotechnology Pvt. Ltd., A-7 M.I.D.C., Mira Industrial Area, Western Express Highway, Mira Road, Thane. Maharashtra – 401 104. INDIA.

[c] Regenerative Medicine Laboratory, Department of Zoology, School of Life Sciences, Periyar University, Salem, Tamil Nadu 636011, INDIA.

[d] Vice Chancellor, Central University of Tamil Nadu, Thiruvarur, Tamil Nadu 610005, INDIA.

[*]Correspondence: nrkumar@mum.amity.edu (Dr. Neelamegam Ramesh Kumar)

Table of Contents

Supplementary figures

Figure S1. Chromatographic gradient–dependent peptide sampling assessed using BSA controls.

Figure S2. Digestion-dependent system suitability performance under the optimized 112-min LC method.

Supplementary tables

Table S1 (XLSX). Historical 111-min gradient, RPM digestion, trypsin 1:20, 4 h.

Table S2 (XLSX). Historical 111-min gradient, NRPM digestion, trypsin 1:20, 4 h.

Table S3 (XLSX). Historical 111-min gradient, RPM digestion, trypsin 1:200, 4 h.

Table S4 (XLSX). Historical 111-min gradient, NRPM digestion, trypsin 1:200, 4 h.

Table S5 (XLSX). Gradient 84-min, RPM digestion, trypsin 1:20, 3 h.

Table S6 (XLSX). Gradient 111-min, RPM digestion, trypsin 1:20, 3 h.

Table S7 (XLSX). Gradient 178-min, RPM digestion, trypsin 1:20, 3 h.

Table S8 (XLSX). Gradient 112-min, RPM digestion, trypsin 1:20, 3 h.

Table S9 (XLSX). Digestion 112-min gradient, RPM digestion, trypsin 1:20, 3 h.

Table S10 (XLSX). Digestion 112-min gradient, NRPM digestion, trypsin 1:20, 3 h, R+A.

Table S11 (XLSX). Digestion 112-min gradient, NRPM digestion, trypsin 1:100, 3 h.

Table S12 (XLSX). Digestion 112-min gradient, NRPM digestion, trypsin 1:100, 16 h.

Table S13 (XLSX). Peptide-level retention time and signal metrics for bracketed SST injections.

Table S14 (XLSX). Comparison of peptide detectability between bracketed SST and matrix-spiked SST.

Table S15 (XLSX). Peptide-level XIC ratio distributions for bracketed and matrix-spiked SST.

Table S16 (XLSX). Subcellular localization annotation of HCPs identified across digestion workflows under the optimized 112-min LC–MS method.

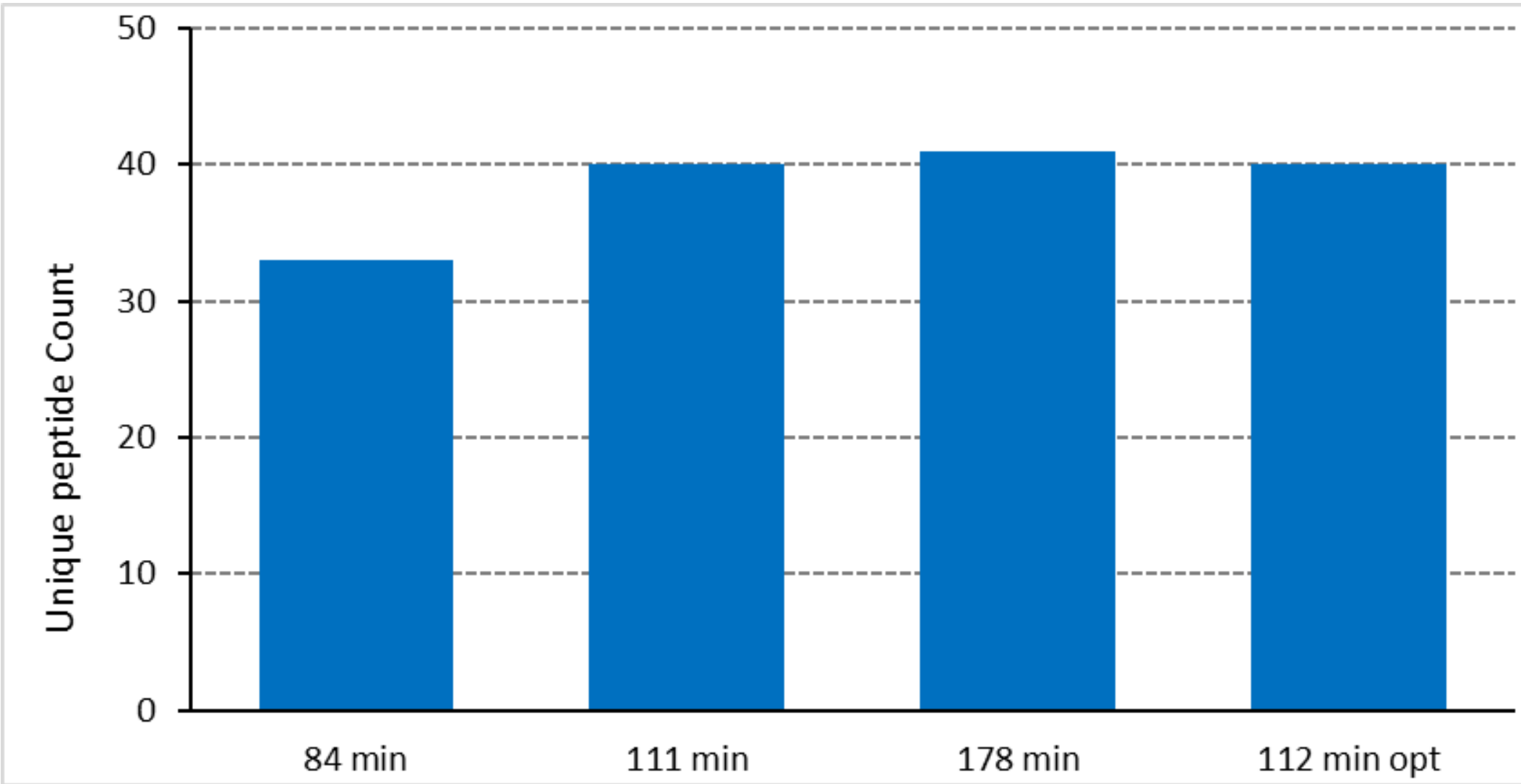

Figure S1. Chromatographic gradient–dependent peptide sampling assessed using BSA controls. Unique peptide recovery for an in-house digested BSA sample analysed under different LC gradient architectures. The compressed 84-min gradient yielded fewer unique peptides, whereas the 111-min, 178-min and optimized 112-min gradients produced comparable peptide recoveries.

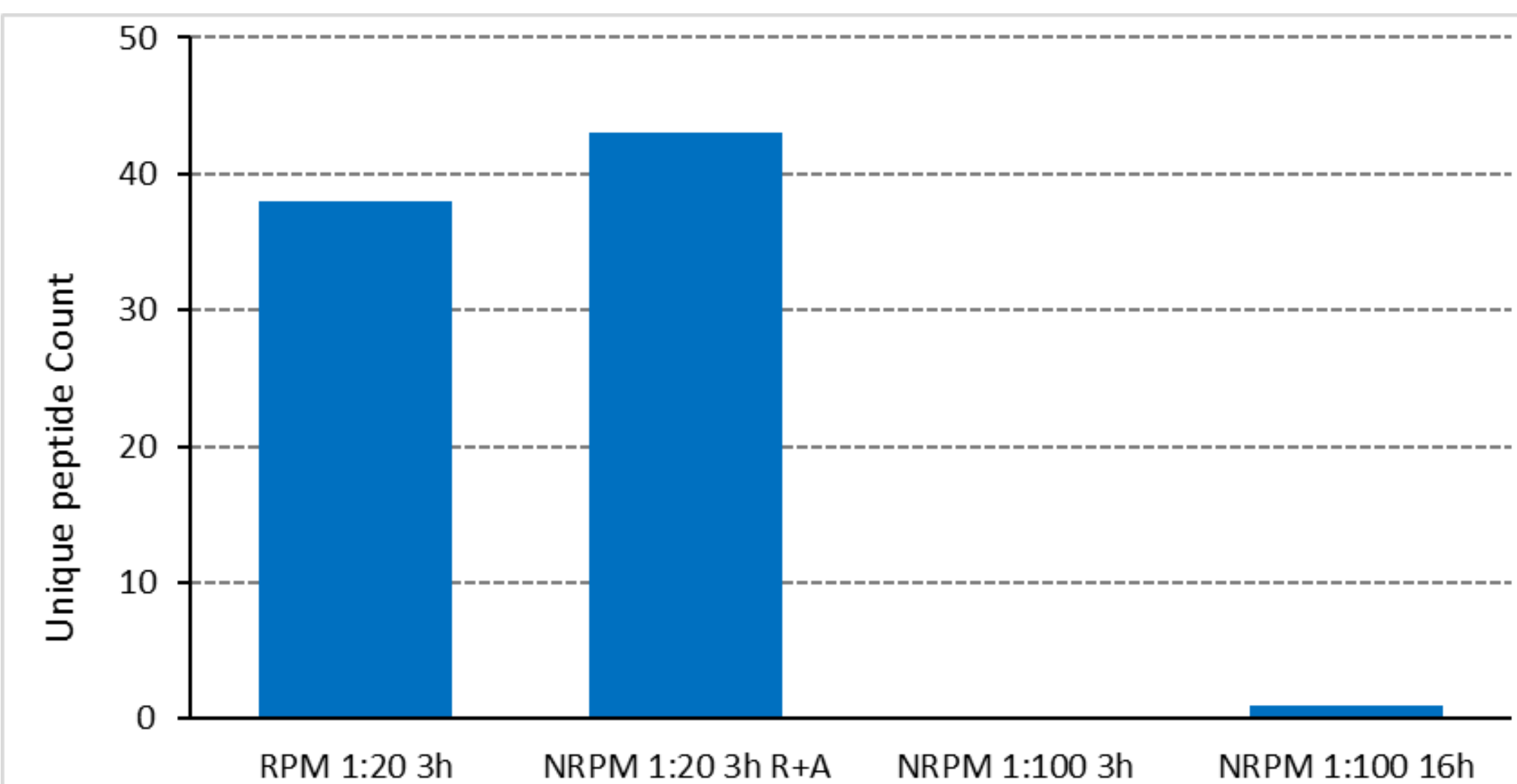

Figure S2. Digestion-dependent system suitability performance under the optimized 112-min LC method. Unique peptide recovery for matrix-spiked BSA across digestion workflows. High-enzyme workflows produced robust peptide recovery, whereas low-enzyme native digestion yielded minimal detectable peptides, with only limited recovery upon extended incubation.
